# A Molecular Toolbox to Identify and Quantify Grape Varieties: On the Trace of “Glera”

**DOI:** 10.3390/foods12163091

**Published:** 2023-08-17

**Authors:** Ilaria Carrara, Valeria Terzi, Roberta Ghizzoni, Stefano Delbono, Giorgio Tumino, Manna Crespan, Massimo Gardiman, Enrico Francia, Caterina Morcia

**Affiliations:** 1 Consiglio per la Ricerca in Agricoltura e l’Analisi dell’Economia Agraria, Centro di Ricerca Genomica e Bioinformatica (CREA-GB), Via San Protaso 302, 29017 Fiorenzuola d’Arda, Italyroberta.ghizzoni@crea.gov.it (R.G.); stefano.delbono@crea.gov.it (S.D.); caterina.morcia@crea.gov.it (C.M.); 2 Plant Breeding, Wageningen University and Research, Droevendaalsesteeg 1, 6708 PB Wageningen, The Netherlands; giorgio.tumino@wur.nl; 3 Consiglio per la Ricerca in Agricoltura e l’Analisi dell’Economia Agraria, Centro di Ricerca Viticoltura ed Enologia (CREA-VE), Viale 28 Aprile 26, 31015 Conegliano, Italy; manna.crespan@crea.gov.it (M.C.); massimo.gardiman@crea.gov.it (M.G.); 4 Department of Life Science, Centre BIOGEST-SITEIA, University of Study of Modena and Reggio Emilia, Via Amendola, n. 2, 42122 Reggio Emilia, Italy; enrico.francia@unimore.it

**Keywords:** chip digital PCR, high-throughput SNP genotyping, varietal traceability, grape chain authenticity

## Abstract

A pillar of wine authenticity is the variety/ies used. Ampelographic descriptors and SSR markers, included in several national and international databases, are extensively used for varietal identification purposes. Recently, SNP markers have been proposed as useful for grape varietal identification and traceability. Our study has been directed toward the development of a molecular toolbox able to track grape varieties from the nursery to the must. Two complementary approaches were developed, exploiting SNP markers with two different technologies, i.e., a high-throughput platform for varietal identification and a digital PCR system for varietal quantification. As proof-of-concept, the toolbox was successfully applied to the identification and quantification of the “Glera” variety along the Prosecco wine production chain. The assays developed found their limits in commercial, aged wines.

## 1. Introduction

Today, wine plays an important role in many cultures throughout the world, serves as a form of enjoyment in others, and is the beverage of choice for those who believe it has health benefits [1,2]. Contrary to many modern foods, wine’s appeal is based on a subtle variety of shifting sensations that make it challenging to pin down. In essence, winemakers are offering their customers a sensory experience. The definition of excellence used to be the exclusive domain of wine producers, and those who did not appreciate a certain style of wine were frequently made to feel uncultured. However, because of globalization and the resulting quick access to information worldwide, consumers are now more informed and powerful, with a more sophisticated grasp of product value and a discriminating desire for quality. Thus, the consumer now has influence over how quality is defined. The varietal composition of a wine is a pillar in this regard.

The Italian wine industry is one of the oldest and most significant in the world. Italy is home to a vast number of grape varieties, many of which are autochthonous and have been cultivated for centuries. The Italian National Register of Grapevine Varieties lists more than 600 wine grape varieties, and there are many autochthonous grapes that make up the Italian grape germplasm, which is distinguished by a high level of variety richness. The Italian wine industry is rich and diversified. It is based on a wide range of grape varieties and offers a very large selection of wines. Among the major wines of the Italian territory, Prosecco is certainly one of the most popular and appreciated in recent years. It is a sparkling wine that has gained a lot of attention due to its fruity and refreshing taste. The wine is produced only in the Veneto and Friuli-Venezia Giulia regions of Italy, where the “Glera” grapes are mainly cultivated. Prosecco wine production is regulated by strict quality control measures to ensure that the wine meets certain standards. One important aspect of Prosecco production is the grape varieties used.

“Glera” is the main grape variety (at least 85%) used in the production of Prosecco, but other local varieties (“Verdiso”, “Bianchetta”, “Glera lunga”, “Perera”) and international varieties (‘Chardonnay’, ‘Pinot blanc’) grown in the area can also be used in smaller quantities.

Wine fraud is a persistent issue in the wine industry, and it can have serious implications for consumers, producers, and the market. Fraud can occur at various stages of the production process, from the vineyard to the bottle, and it can involve different forms of misrepresentation, such as counterfeiting, adulteration, mislabeling, or theft. The motives for fraud can vary, from financial gain to prestige or reputation, and they can involve different actors, from rogue operators to organized crime groups.

The consequences of wine fraud can be severe. For consumers, it can mean paying a premium for a fake or low-quality product, as well as disappointing sensory experiences. For producers, it can mean losing market share and incurring sanctions. For the market, it can mean a loss of trust, a decrease in demand, and a distortion of price signals.

To prevent and detect wine fraud, traceability and identity preservation are critical [3,4]. Traceability refers to the ability to track the movement of a product or its components from the source to the destination using unique identifiers and documentation. Identity preservation refers to the ability to maintain the integrity and authenticity of a product or its components throughout the production process using appropriate protocols and technologies.

In the wine industry, traceability can be achieved through a variety of methods, such as unique identification codes, barcoding, and electronic tracking systems. These methods can help ensure that wines are correctly labeled and that their authenticity can be verified at every stage of the supply chain.

Identity preservation in the wine industry involves maintaining the distinct characteristics and quality of each wine. This can be achieved through strict regulations on production methods, grape varieties, and geographical indications. For example, the European Union has established the Protected Designation of Origin (PDO) and Protected Geographical Indication (PGI) labels, which provide legal protection for traditional and regionally produced products.

The grape plant is therefore the starting point of wine authenticity, both in terms of the grape variety used and of the plant cultivation area. The geographical origin is one of the most important factors that determine the commercial value and is a key point in the defense of PDO and PGI products against producers outside the designated area. Isotopic analysis can measure the chemical signatures of wines, which can reveal their geographical origin or production history. Geochemical markers have been developed for wine authenticity assessment, as reviewed by Camin et al. [5]. Petrini et al. [6] successfully applied the Sr-isotopic systematics to must from different “Glera” vineyards in the Veneto Region (Italy), whereas Pepi and Vaccaro [7] developed geochemical fingerprints of Prosecco wine based on major and trace elements.

In addition to geographical origin, another pillar of wine authenticity is the variety/ies used. As reviewed by Villano et al. [8], the cultivated grapevine includes an enormous population of varieties and clones shaped by human and environmental selection over centuries of cultivation, by gametic and somatic mutations, and by sexual and asexual propagation. The need to have an effective characterization system essential to defining the varietal identity and avoiding cases of synonymy and homonymy was highlighted early. Starting from ampelographic and phenotypic characterizations [9], a plethora of DNA-based technologies are currently available. Among these, SSRs are now the golden standard, widely used and shared worldwide. For varietal fingerprinting, the international scientific community has selected and proposed sharing sets of SSRs [10]. Their profiles are included in the major dedicated grape databases, both international (such as the *Vitis* International Variety Catalogue (VIVC) [11] and the European Vitis Database [12]) and national (such as the Italian Vitis Database [13], the Swiss Vitis Microsatellite Database [14], and the Portuguese Vitis Database [15]). Furthermore, a panel of SNP profiles was recently included in the VIVC database. Emerging tools are sets of SNPs derived from the sequencing data of several grapevine genomes [16]. Such molecular markers are the protagonists of a vast field of study in grapevine, which goes from the study of the domestication of this plant to the determination of the genetic relationships between accessions up to the study of the genealogy of specific grape varieties [17,18,19,20].

Recently, SNP markers have been proposed as useful for grape varietal identification and traceability [8]. The main advantages of these markers are that they occur in genomes at a much higher frequency than SSRs and that they can be genotyped in high-throughput systems with different multiplex ratios. Among the methods allowing medium SNP multiplexing (from tens to hundreds) with high throughput, there is the BioMark (Fluidigm, San Francisco, CA, USA) platform, based on microfluidic chips consisting of a network of capillary channels, valves, and reaction chambers [21]. Samples and assays are distributed within the chip in such a way that each of the samples loaded into the sample inlets is assayed for each of the assays loaded into the assay inlets. This system has been exploited in several grape studies, among them grapevine defense status evaluation [22], QTL mapping [23], genetic variability determination [24], and gene expression studies [25].

In our study, the applicability of this technology for varietal traceability in grapes has been evaluated. Moreover, digital PCR assays have been developed for varietal quantification.

A ground-breaking technique for sensitive and precise nucleic acid measurement is, in fact, digital PCR (dPCR). This is a third-generation method for the amplification of nucleic acids. The division of the sample into several distinct compartments, each of which has an independent amplification reaction, is a distinctive aspect of the approach. For this goal, several instrumental platforms have been created, and a variety of statistical methodologies are available for reading the digital output data. With the use of microfluidics, the sample is divided into several partitions (hundreds, thousands, or, on some platforms, even millions of partitions). Each partition behaves as a separate reaction, is subjected to a typical PCR, and is then scored as positive or negative. The initial target concentration is calculated by exploiting the Poisson distribution as the ratio of positive partitions to all counted partitions. The uses, benefits, drawbacks, and applicative perspectives of the technology in plant science have been reviewed by Demeke and Dobnik [26] and by Morcia et al. [27].

In sum, our study has been directed toward the development of a molecular toolbox able to track grape varieties from the nursery to the must. Two complementary approaches were developed, exploiting SNP markers with two different technologies, i.e., a high-throughput platform for varietal identification and a digital PCR system for varietal quantification. As proof-of-concept, the toolbox was applied to the identification and quantification of the “Glera” variety along the Prosecco production chain.

## 2. Materials and Methods

### 2.1. Materials

Twenty-four “Glera” accessions and 136 grapevine varieties were used. The 24 “Glera” accessions were recovered over the years in the traditional growing area of the variety and conserved in the CREA germplasm repository. The 136 grapevine samples (Table S1) were derived from a wider collection of 615 varieties hosted in various Italian collections, including 343 from the CREA Research Centre for Viticulture and Enology in Conegliano, 79 from local collections in Piedmont managed by the Italian National Research Council and the University of Turin, 54 from collections in Emilia-Romagna and Lazio managed by the University of Modena and Reggio Emilia and the University of Tuscia, 90 from collections in Tuscany managed by the University of Pisa, 27 from collections in Apulia managed by the University of Foggia, and 22 from collections in Calabria and Sicily managed by the University of Palermo.

Three different commercial wines were purchased. All carried the denomination Prosecco DOCG and were selected based on their costs: low, medium, and high.

### 2.2. DNA Extraction

DNA was extracted from “Glera” in different matrixes, i.e., leaves, berries, and must, and from leaves of the 136 grapevine accessions using a CTAB-based buffer followed by chloroform extraction (Merck Life Science, Milano, Italy) [28]. The evaluation of the quality and quantity of the extracted DNA was performed using a Qubit™ fluorometer in combination with the Qubit™ dsDNA BR Assay kit (Invitrogen by Thermo Fisher Scientific, Monza, Italy).

Four different extraction methods were used for DNA extraction from the commercial wines [15,16]. The four methods are briefly reported below.

- 1st method: sodium chloride (Merck Life Science, Milano, Italy) precipitation-based, according to Savazzini et al. [29].

A total of 45 mL of sample was added to 5 mL of NaCl (1.2 M) (Merck Life Science, Milano, Italy). The solution was homogenized and stored at −20 °C for at least a week.

- 2nd method: isopropanol (Merck Life Science, Milano, Italy) precipitation-based, according to Savazzini et al. [29].

A total of 20 mL of sample was added to 0.7 volumes of isopropanol (Merck Life Science, Milano, Italy). The solution was homogenized and stored at −20 °C for at least two weeks.

- 3rd method: sodium acetate (Merck Life Science, Milano, Italy) precipitation-based, according to Savazzini et al. [29].

A total of 43 mL of sample was added to 7 mL of sodium acetate (3 M) at 5.2 pH (Merck Life Science, Milano, Italy). The solution was homogenized and stored at −20 °C for at least two weeks.

- 4th method: according to Zambianchi et al. [30].

A total of 40 mL of wine was combined with 1 volume of isopropanol (Merck Life Science, Milano, Italy) and 0.3 volumes of sodium acetate (3M) at pH 5.2 (Merck Life Science, Milano, Italy). The mixture was refrigerated at −20 °C for at least 2 days. After that, the samples were centrifuged (8500 rpm) for at least 3 h at 4 °C. After removing the supernatant, the pellet was resuspended in 500 microL of isopropanol (Merck Life Science, Milano, Italy) and refrigerated at −20 °C for 2 days to aid in DNA precipitation.

### 2.3. High-Throughput SNP Genotyping

The analysis was carried out using the Fluidigm Biomark™ HD automated, high-performance PCR system. The 192.24 IFC was employed, which allowed the inlet of 192 samples and 24 assays that underwent amplification in a total of 4608 reaction chambers.

The IFC was loaded with the SNP Type Assay Mixes and Sample Mixes, both of which were prepared according to Fluidigm’s manufacturer’s instructions.

The SNP Type Assay Mixes preparation consisted of the following workflow:

- 24 μL of SNP Type Assay ASP1/ASP2 (100 μM each);
- 64 μL of SNP Type Assay LSP (100 μM each);
- 700 μL of DNA suspension buffer.

The total was dispensed into 24-plate wells using a multichannel pipette.

The 10X Assay preparation consisted of the following workflow:

- 48 μL of 2X Assay Loading Reagent (Fluidigm PN 100-7611);
- 29 μL of PCR-certified water.

The total was dispensed into the 24-plate wells already containing the SNP Type Assay Mixes.

Sample Mixes were constituted of 1.9 μL of grape DNA at 20 ng/μL added to 2.6 μL of a sample pre-mix made of:

- 472.5 μL of Biotium 2X Fast Probe Master Mix (Biotium, PN 31005);
- 47.25 μL of 20X SNP Type Sample Loading Reagent (Fluidigm, PN 100-3425);
- 15.75 μL of 60X SNP Type Reagent (Fluidigm, PN 100-3402);
- 5.67 μL of ROX 50X (Life Technologies, PN 12223-012) at 25 μM;
- 10.08 μL of PCR-certified water.

### 2.4. SNP Selection for Fluidigm Biomark™ HD Genotyping

The genotyping was carried out based on the 46 KASP markers proposed by Wang et al. [31]. The KASP assays were optimized for the Biomark platform. The optimization, realized using Standard BioTools (San Francisco, CA, USA), included the removal of KASP tags, the addition of SNP Type tags for the detection primers, and the reutilization of the common primers. Table S2 reports the SNP Type^TM^ Assays used.

The data obtained after SNP genotyping were filtered for quality. Neighbor-joining clustering analysis was carried out using the PAST 4.03 software package, freely available at folk.uio.no/ohammer/past and updated from the original version developed by Hammer et al. [32]. The Jaccard similarity index (cophenetic correlation of 0.7773) was used. Polymorphic information content (PIC) and Heterozygosity (H) values were calculated using the freely available online tool https://gene-calc.pl/pic.

### 2.5. SNP Selection for Digital PCR Analysis

The SNP dataset developed by D’Onofrio et al. [28] was exploited to select “Glera”-specific markers. The dataset includes 615 grapevine accessions genotyped using the Infinium 18K Grape Array (Illumina Inc., San Diego, CA, USA), as reported in D’Onofrio et al. (2021). Array signals were converted into discrete genotypes using GenomeStudio (Illumina Inc., San Diego, CA, USA). The same technological approach was used to genotype the 24 “Glera” clones.

The following selection steps were used to identify useful SNPs:

- Only homozygous markers without any missing values in all the genotypes were considered;
- In the reduced SNP panel obtained after step (a), allelic variants almost unique to “Glera” were individuated;
- The markers individuated in step (b) were checked to be in the same allelic status in all the “Glera” accessions;
- The selected candidate SNPs were mapped on the grapevine genome sequence using BLAST (Basic Local Alignment Search Tool).

**Figure 1:**
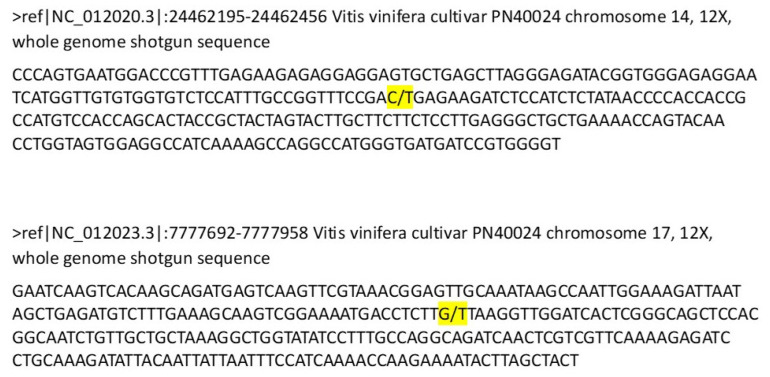
Grape genomic regions carrying polymorphisms (highlighted in yellow) on which the two digital PCR-based assays ID AN33KY7 and ID AN49FJ4 aimed at “Glera” traceability were designed.

Two SNPs mapped on chromosomes 17 and 14 were selected for further analyses (Figure 1).

### 2.6. Chip Digital PCR Assay

Primers and MGB probes were designed on the SNP sequences (Figure 1) using the Custom TaqMan^®^ SNP Genotyping Assay procedure (Thermo Fisher Scientific, Monza, Italy) and are available as assays ID AN33KY7 and ID AN49FJ4 (Catalog n. 4332077, Thermo Fisher Scientific, Monza, Italy). In the dPCR assay ID AN33KY7, the “Glera” target allele was marked with VIC, whereas the alternative, non-“Glera” allele was marked with FAM. In the dPCR assay ID AN49FJ4, the “Glera” target allele was marked with FAM, whereas the alternative, non-“Glera” allele was marked with VIC.

Chip digital PCR was performed using the QuantStudioTM 3D Digital PCR System (Applied Biosystems by Life Technologies, Monza, Italy). The reaction mixture was prepared in a final volume of 16 µL consisting of 8 µL QuantStudioTM 3D Digital PCR 2X Master Mix, 0.4 µL of Custom TaqMan^®^ SNP Genotyping Assay 40X (Catalogue number 4332077, Applied Biosystems by Life Technologies, Monza, Italy) containing primer and VIC/FAM-MGB probes, 1 µL of DNA (5 ng/µL), and nuclease-free water. In addition, a negative control with nuclease-free water as a template was added. A total volume of 15 µL of reaction mixture was loaded onto the QuantStudioTM 3D Digital PCR chips using the QuantStudioTM 3D Digital chip loader, according to the manufacturer’s protocol.

Amplifications were performed in a ProFlexTM 2Xflat PCR System Thermocycler (Applied Biosystems by Life Technologies, Monza, Italy) under the following conditions: 96 °C for 10 min, 47 cycles of 60 °C annealing for 2 min, and 98 °C denaturation for 30 s, followed by 60 °C for 2 min. End-point fluorescence data were collected in a QuantStudioTM 3D Digital PCR Instrument, and the files generated were analyzed using cloud-based platform QuantStudioTM 3D AnalysisSuite dPCR software, version 3.1.6. Each sample was analyzed in triplicate.

## 3. Results

### 3.1. SNP Genotyping

The assay panel reported above was used to genotype 136 grape accessions, many of which are listed in the Italian National Register. A total of 8 assays were filtered out because of their lower amplification efficiency level on the Biomark platform, and a core set of 38 assays was retained as suitable for high-throughput system-based genotyping The 38 markers univocally identified 98% of the 136 accessions.

The PIC and H values were calculated for the 38 codominant markers used in the tested varieties [33] with the aim of evaluating their informativeness. Among the 38 markers considered, three classes were found:

- A total of 22 markers have a PIC > 0.5 and a mean H value of 0.61.
- A total of 11 markers have a PIC between 0.5 and 0.4 and a mean H value of 0.54.
- A total of 5 markers have a PIC between 0.4 and 0.3 and a mean H value of 0.43.

**Figure 2:**
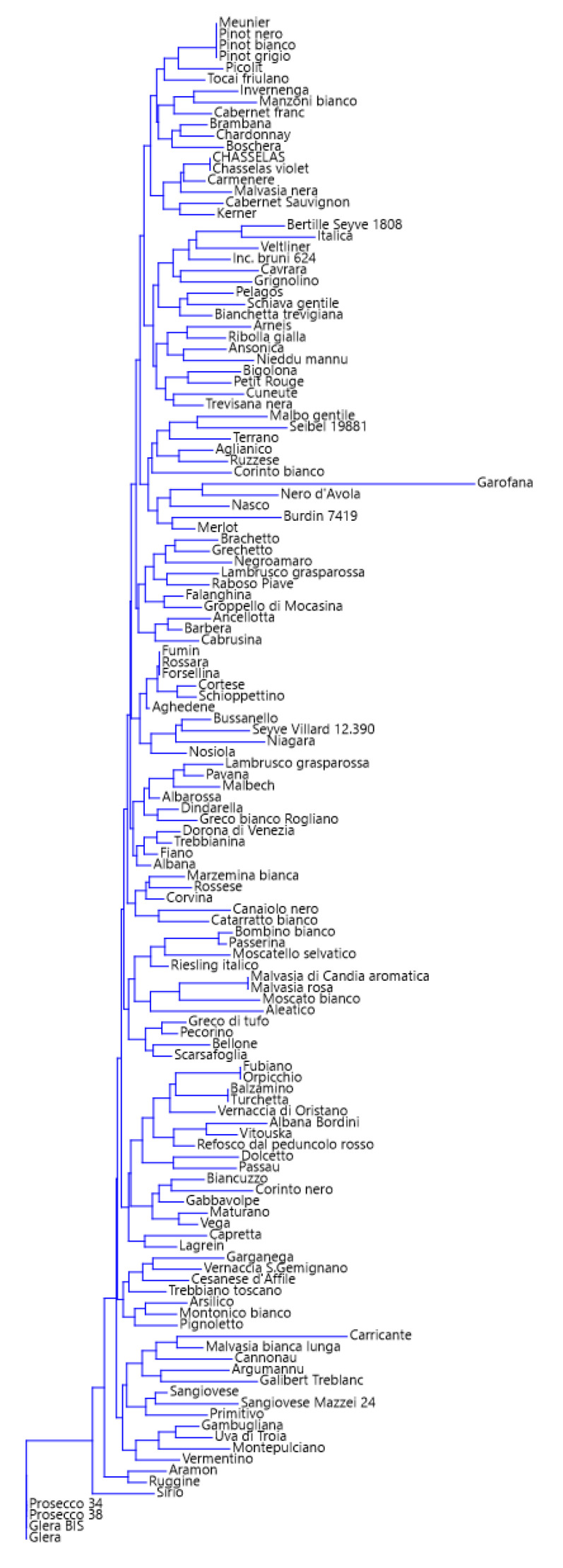
“Glera”-rooted dendrogram obtained after neighbor-joining clustering analysis.

Because, among the 38 markers considered in this study, 22 can be considered very informative, the efficiency of this reduced set for grape varietal identification was evaluated. The phenetic analysis was repeated on the whole set of 136 varieties using the core set of 22 markers, selected based on PIC > 0.5. Figure 2 shows the dendrogram obtained after clustering.

The 22 markers univocally identified 96.4% of the 136 accessions. In comparison with the initial set of 38 markers, the efficiency was therefore slightly reduced (minus 1.6%), but the possibility of using fewer markers is of interest because it correlates to the reduction in analytical costs. On the basis of the results obtained, it can be concluded that even the reduced set of 22 markers can quite satisfactorily be used for grape varietal identification.

### 3.2. Digital PCR

The workflow adopted included the following steps:

1. Screening of an SNP database developed after genotyping 615 grape varieties with an 18K Infinium SNP array;
2. Identification of “Glera”-informative SNPs;
3. Validation of the selected SNPs on a panel of “Glera” accessions;
4. Development of chip digital PCR assays designed for the selected SNPs;
5. Evaluation of assay performance along the Prosecco production chain.

Two SNPs (Figure 1) were selected as informative to track “Glera”:

- A transition C/T in position 24462302 of grape chromosome 14, with “Glera” carrying the C allele;
- A transversion G/T in position 7777803 of grape chromosome 17, with “Glera” carrying the T allele.

#### 3.2.1. Digital PCR Assay Specificity

The specificity of the two assays was evaluated at two levels:

- Among “Glera” accessions;
- Among “Glera” and non-“Glera” varieties.

Somatic mutations and epigenetic effects are the main sources of intra-varietal diversity expressed by clones [34,35,36,37]. Twenty-four “Glera” clonal variants were used to evaluate the ability of the assays to track “Glera” variants. “Glera” and all its variants, genotyped with the 18K SNP Infinium array, share the same allelic status in the chromosomal sequences selected for the assay design; therefore, the two assays developed are not affected by intra-varietal variability. On the contrary, all the 615 non-“Glera” varieties considered have an allelic status in the chromosomal sequences selected for the assay design different from “Glera”, with very few exceptions. The exceptions are the “Cannonau” and “Vitouska” varieties, which share the same allelic status as “Glera” in both loci considered and are therefore not distinguishable from “Glera” with the two combined dPCR assays developed. Therefore, the probability of a mixed harvest of the two grapes is remote. To the best of our knowledge, no genetic relationships have been found between “Cannonau” and “Glera” [38]. On the contrary, SSR and SNP data support the hypothesis that “Vitouska” has parental relationships with “Glera” [39,40]. Moreover, “Vitouska” is grown in the Kars region and therefore shares the cultivation area with “Glera”. However, “Vitouska” is a minor white wine grape variety, recently recovered and revalued by local viticulturists, grown in small parcels.

**Figure 3:**
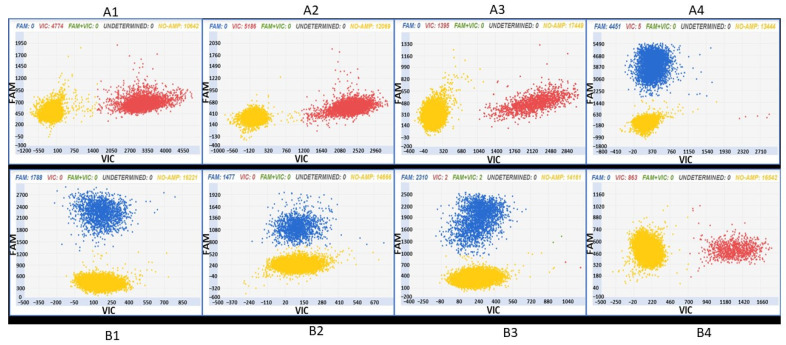
Chip digital PCR analysis of three “Glera” accessions (**A1**–**A3**; **B1**–**B3**) and one non-“Glera” variety (**A4**,**B4**) with the two assays ID AN33KY7 (samples A1–4) and ID AN49FJ4 (samples B1–4). Samples A1–3, in which 100% “Glera” DNA was added as a template, show a VIC signal, visualized as red dots (the “Glera” allele was marked with VIC), whereas the A4 sample (i.e., non-“Glera” variety DNA) gave a FAM signal (blue dots). Samples B1–3, in which 100% “Glera” DNA was added as a template, show a FAM signal, visualized as blue dots (the “Glera” allele was marked with FAM), whereas the B4 sample (i.e., non-“Glera” variety DNA) gave a VIC signal (red dots). All of the patterns have a yellow cloud due to DNA-empty wells.

In Figure 3, examples of the analytical output obtained after the digital PCR analysis of the “Glera” and non-“Glera” varieties with the two assays are reported.

The level of specificity is such as to ensure that all “Glera” accessions are distinguished from 98.6% of the 615 grapevine varieties considered in this study based on the SNP assay designed for chromosome 14 and from 97% of the 615 grapevine varieties considered in this study based on the SNP assay designed for chromosome 17. The combination of both assays can distinguish “Glera” from 613 out of the 615 varieties considered. In fact, as already reported, only “Cannonau” and “Vitouska” share the same allelic status as “Glera” in the two loci exploited for the assay design and are therefore not distinguishable among them.

#### 3.2.2. Digital PCR Assay Precision, Trueness, and Applicability

The precision of the assays, i.e., the closeness of agreement between replicated measurements, was determined on four “Glera” samples in double replicates, and the mean precision was found to be 7.5%, which is below the 35% limit fixed as acceptable, according to the Codex Alimentarius Commission/Guidelines 74–2010 [41].

**Figure 4:**
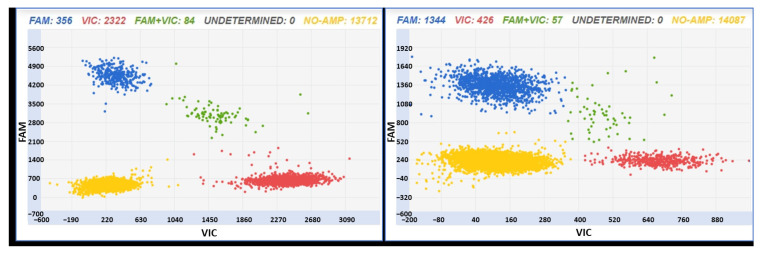
Two-dimensional scatter graphs generated by chip digital PCR analysis of “Glera” and non-“Glera” mixed DNA (85% “Glera” and 15% non-“Glera”) with the two assays ID AN33KY7 (left panel) and ID AN49FJ4 (right panel). In the left panel, red dots stand for “Glera” and blue dots for non-“Glera” varieties. On the other hand, in the right panel, blue dots stand for “Glera” and red dots for non-“Glera” varieties. All of the patterns have a yellow cloud due to DNA-empty wells.

The trueness of the assays was evaluated on samples obtained by mixing DNA extracted from “Glera” and non-“Glera” varieties (Figure 4). The samples were prepared by mixing 85% “Glera” DNA and 15% non-“Glera” DNA and analyzed with the two assays ID AN33KY7 and ID AN49FJ4. The chosen percentages are consistent with the Prosecco production disciplinary, which requires a minimum “Glera” content of 85%.

The trueness of the method is usually defined as the degree of agreement of the expected value with the true value or accepted reference value. In GMO testing, the trueness must be within 25% of the accepted reference value [42]. The trueness of the assays for “Glera” tracking fits the purpose: the estimated concentrations over the dynamic range of interest according to the Prosecco production disciplinary were within the ±25% acceptable bias, as recommended by the GMO analytical guidelines [42].

**Figure 5:**
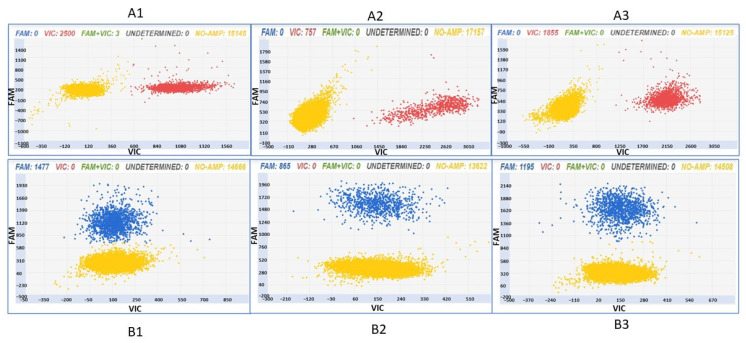
Two-dimensional scatter graphs generated by chip digital PCR analysis of DNA extracted from leaves (**A1**,**B1**), berries (**A2**,**B2**), and musts (**A3**,**B3**) of “Glera” with the two assays ID AN33KY7 (samples A1–3) and ID AN49FJ4 (samples B1–3).

The applicability of the assays was evaluated on a panel of “Glera” samples, i.e., leaves, berries, and musts. The two assays were found useful to track “Glera” in leaves, berries, and musts (Figure 5). Samples A1–3 show VIC signals, and samples B1–3 show FAM signals. All the patterns have a yellow cloud due to DNA-empty wells. The two digital PCR assays can therefore be applied to identify “Glera” in nursery plants, during berry storage, and in the first steps of vinification.

**Figure 6:**
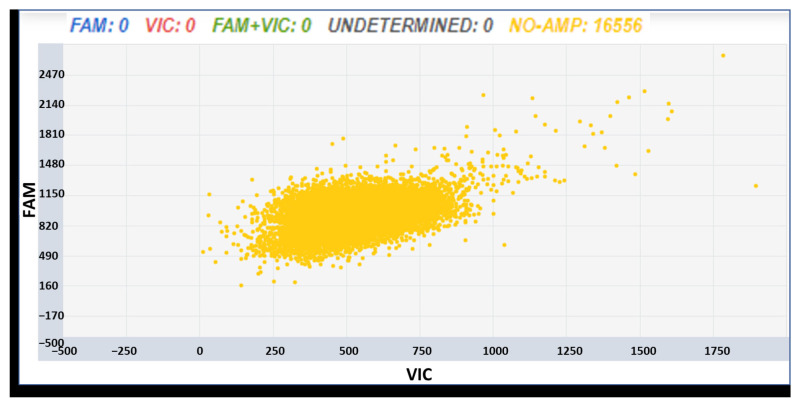
Output of a dPCR analysis carried out with the assay ID AN49FJ4 on extracts from Prosecco commercial wine sample. Yellow dots stand for no amplification.

The possibility of applying dPCR assays developed for commercial wines was evaluated. A panel of Prosecco bottles was purchased from a local market and subjected to DNA extraction using several different approaches, as reported in the Material and Methods section. The extracts were analyzed with both dPCR assays, and an example of a dPCR plot is reported in Figure 6. No amplifications were obtained; a failure in the possibility of tracking the varietal content in such matrices due to the absence of amplifiable DNA was found.

## 4. Discussion

In our study, two different molecular approaches have been implemented, both based on SNPs but exploiting different technologies, i.e., microfluidic array and digital PCR, with different aims, namely varietal identification and varietal quantification, respectively.

For varietal identification, our study built on the SNP set proposed by Wang et al. [31], applicable to the identification of both varieties and rootstocks, further evaluating its application with a high-throughput platform. As a first step, a set of 38 SNPs was used for varietal fingerprinting. The set can univocally identify the grape varieties considered, with a few exceptions. The three varieties “Fumin”, “Forsellina”, and “Rossara” are in fact clustered together and cannot be distinguished from each other. The “Glera” accessions are correctly clustered together and are univocally identified among the 136 varieties considered. Also noteworthy is the fact that “Chasselas” and “Chasselas violet”, which are different in berry color but very close from a genetic point of view, are distinguishable from each other based on one of the analyzed SNPs. This same SNP can discriminate between “Malvasia aromatica di Candia” and “Malvasia rosa”, which differ in berry color but are genetically very close to each other. This SNP falls within the *Vitis vinifera* ABC transporter C family sequence, coding for transmembrane transport proteins. Biochemical, molecular, and genetic evidence support the involvement of the ABCC proteins in the transport of anthocyanins [43,44]. This is only an indication, which could be better investigated to understand if the identified polymorphism can have an effective role in conferring the different color in accessions with the same genetic heritage.

As a further step, a core set of 22 SNPs was identified as useful for fingerprinting. The difference in resolution between the 22-marker core set and the entire set of 38 markers lies in the inability to distinguish the two varieties “Sirio” and “Italica”. In the dendrogram of Figure 2, the two varieties “Sirio” and “Italica” are in fact clustered together. It is noteworthy that these two accessions are the result of two breeding programs that used the “Verdiso” genotype as one of the parents. According to the VIVC database, in fact, “Italica” is the result of “Verdiso” X “Welschriesling”, whereas “Sirio” has been obtained by crossing “Verdiso” X “Madeleine Royal”.

A similar reduction in marker number was proposed by Wang et al. [31] on 348 grape varieties: from the initial set of 46 SNP markers, a core set of 25 markers was individuated as useful for fingerprinting. Such a core set of 25 KASP markers could in fact distinguish 95.69% of the 348 varieties tested, and the other 21 markers were proposed as extended markers. The core set markers selected by Wang et al. [31] are partially overlapping with the core set individuated in the present study: 13 markers are in fact shared by both studies, whereas 9 markers belong to the core set in our study and to the extended set in the study of Wang et al. [31]. The differences in the performance of the same markers found in the two studies can be explained first of all by the different grape germplasm considered in the two studies and even by the different technological approaches used, i.e., KASP PCR products detected by a fluorescent microplate detector in the study of Wang et al. [31] and SNP Type PCR products detected in a Biomark Fluidigm platform in this study. A common feature of the two studies is that both are based on SNP detection technologies different from gene chip technology. This last is dedicated to multi-site detection since tens of thousands of allelic variations can be identified with gene chip technology. However, the development and validation of a chip are long processes, the current cost of its use is relatively high, and the data analysis is more complex. Contrarily, KASP technology is particularly suitable for the detection of multiple samples at a few sites and is efficient, flexible, accurate, and low-cost [45]. Some studies in grapes have adopted this technology, but not specifically for varietal traceability. Calderon et al. [46] genotyped a collection of “Malbec” accessions using 41 Single-Nucleotide Variants with Fluidigm to study the intra-cultivar genetic diversity in grapevines. Nebish et al. [47] profiled the DNA of autochthonous Armenian grapes with a set of 240 nuclear SNP markers applying Fluidigm technology with the aim of identifying accessions as well as providing pedigree information.

To the best of our knowledge, the use of the Fluidigm platform for grape varietal traceability using SNPs is therefore new. In comparison with SSR-based fingerprinting, the main advantage of using SNP markers can be identified in the opportunity for multiplexing, which impacts directly on the analytical time and costs. In fact, hundreds of DNAs can be simultaneously characterized with tens of SNPs in a few hours, and this is the main reason why this approach is cost-effective in comparison with other technologies.

Equally innovative is the use of digital PCR in grape traceability. Digital PCR assays were developed with the aim of being able to confirm the identity of a specific grapevine, i.e., “Glera”, at the level of plants in the nursery and also quantify it in subsequent phases of the grape utilization chain, such as the harvest and the initial phases of berry vinification. According to this objective, the molecular marker database obtained after genotyping 615 grapevine varieties with the Infinium 18K SNP array was screened for target variety diagnostic alleles. As a final deliverable, two cdPCR assays to specifically track and quantify the “Glera” variety were made available. Digital PCR is an innovative tool that can find vast potential applications in the genetic improvement in grapes, the control of their pathogens, and the defense of product quality and authenticity. At the moment, most of the applications concern the diagnosis of pathogens. A bibliographic search using the keywords “digital PCR” and “grapevine” shows how, in the last five years, about 80% of digital PCR applications in the grape–wine supply chain is oriented towards the identification of microorganisms. Few studies have applied this technology to other objectives, such as the evaluation of gene expression or the control of edited plants. Moving toward traceability applications, Morcia et al. [48] developed a digital PCR for the identification and quantification of Muscat-flavored grapes, but no example of digital PCR applied to the traceability of a specific grape variety has been proposed until now. Several DNA-based technologies are currently used for varietal genotyping [8], among them even one able to give in-the-field varietal authentication [49]. However, only real-time PCR and digital PCR can give a quantification of the target variety. To the best of our knowledge, a real-time PCR approach has been proposed only by Boccacci et al. [50] to track the “Nebbiolo” variety. The dPCR assays developed in this study proved to be effective and economical in tracing and quantifying the “Glera” variety and its variants, starting from the plant up to the must.

No amplification signal was obtained using the DNA extracted from the “Glera” commercial wine samples as a template; therefore, our dPCR assays cannot cover the whole Prosecco production chain. The reason for this failure is the quantity of DNA extracted from the wine samples. DNA has in fact been easily extracted from leaves, berries, and musts, but not from wines (Table 1).

This limitation on commercial wine’s applicability is shared by the majority of studies published on this topic. SSR-based analysis has been found to work on varietal traceability in musts and less in wines by several authors [51,52,53,54,55,56,57]. Recupero et al. [52] suggested that, because of the limited quantity of amplifiable DNA in wine, traceability can start primarily in the musts. CpSSR is reported to work better than SSR to track varieties in musts and in experimental wine samples [53,58]. Boccacci et al. [53] found that SSR analysis works on samples obtained after grape crushing and pressing and after static clarification or flotation but is not satisfactory on samples collected halfway through fermentation or on finished wines. Garcia-Beneytez et al. [56] found that genotyping was possible in experimental wine until decanting, when the particles in suspension were removed. Siret et al. [57] confirmed that DNA extraction and the subsequent SSR analysis can be performed in musts and in experimental wines, but the applicability of the technique in commercial wines has severe limitations.

Agrimonti and Marmiroli [51] demonstrated that, by combining CTAB with purification and NucleoSpin Plant Kit columns, it is possible to extract DNA even from commercial wines, even if the success of the analysis also rests on the target SSRs. Moreover, these authors underlined that “*the success of DNA analysis also depends on the number of technical and biological replicates, since even slight variations in extraction can affect the result of PCR*”.

Some positive results have been obtained on wines sampled in the early stages of winemaking. Zambianchi et al. [30,59] evaluated the applicability of SSR analysis on wine sampled in the real case of a large Italian cooperative winery, finding that microsatellite analysis for cultivar identification can be used from the grape to the “young” wines at the end of the most common oenological operations. Contrarily, the aging process after tank storage and bottling can drastically change the scenario. After four months of storage and two months of bottling, the genetic profile obtained was clearly incomplete. Therefore, the capacity to recognize production cultivars was strongly reduced by wine aging [59]. Moreover, some technological treatments can contribute to DNA degradation in wine. High-quality wines intended for widespread marketing, as is the case with Prosecco, need stabilization and clarification. Several oenological products have been allowed for this purpose [42]. In Prosecco production, bentonite is the preferred agent for clarification, followed by vegetal proteins and yeast-derived products, whereas animal gelatine is marginally used. Bentonite treatment, according to Gambino et al. [60], reduces the wine DNA content by 99.56%, vegetal protein-based treatment by 80%, and yeast-based treatment by 78%. Similarly, the commercial Prosecco wine samples analyzed in this study were treated with one or more of these clarification agents, causing an extensive loss of extractable and amplifiable DNA. The failure in the applicability of dPCR assays to commercial Prosecco wines experienced in the present study is therefore explainable based on the findings of Gambino et al. [60] and of Zambianchi et al. [59], consistent with the current literature, and, therefore, expected. However, as suggested by Agrimonti and Marmiroli [51], there is the potentiality that, by increasing the number of technical and biological replicates, DNA can be extracted even from commercial wines, even if this means an increase in the analytical costs.

The greatest barrier to varietal traceability in commercial wine remains its extremely low DNA content, which is correlated with the wine’s processing and aging. This same obstacle is well known in the GMO traceability sector, which has identified several products from which DNA extraction is challenging, as reviewed by Sajali et al. [61]. DNA can be heavily degraded by processing; for example, Mafra et al. [62] reported that a high quantity of good DNA can be extracted from low-processed soybean products, such as tofu or soybean flours, whereas not-amplifiable DNA was retrieved from highly processed soybean sauce. In the case of wine, the impact of different oenological treatments and aging can also explain the variability in the effectiveness of DNA extraction from different wines. Slightly aged and minimally clarified and filtered wines can be possible matrices for extracting amplifiable DNA, unlike aged wines subjected to complex clarification and filtration.

## 5. Conclusions

A molecular toolbox has been developed aimed at the identification and quantification of grape varieties, from the nurseries to the first step of vinification, i.e., the must. Two different molecular approaches have been implemented, both based on SNPs but exploiting different technologies and with different aims. The panel of informative SNP markers conjugated with a high-throughput analytic platform can be exploited in the context of grape germplasm management, ranging from conservation activities to control in plant nurseries. The dPCR assays proved to be effective and economical in tracing and quantifying “Glera” accessions, starting from the plant up to the must. The application of digital PCR assays developed in this study to identify and quantify a grape target variety is a novelty. One of the premises of this study was to evaluate the applicability of such a technique along the Prosecco production chain, taking into consideration that dPCR is less sensitive to PCR inhibitors in comparison with other methods [12]. Moreover, the dPCR assays developed produce small amplicons, a feature compatible with fragmented DNA templates. However, the difficulty of applying the analytical system to aged commercial wines is to be verisimilarly referred to by the almost total absence of DNA from the samples, as reported by the authors who have investigated the wine aging phase and the oenological treatments [50,59,60].

## Figures and Tables

**Table 1:** DNA quantity and quality extracted from leaves (500 mg), berries (500 mg), and musts (20 mL). The total DNA extracted was resuspended in 50 µL final volumes. The minimum and maximum values of DNA quantity (expressed as ng/µL) and of DNA quality (expressed as 260/280 nm) among replicated extractions are reported.

Samples	ng/µL DNA (Final Volume: 50 µL)	260/280 nm
Leaves	39–63	1.72–1.95
Berries	7–18	1.98–2.21
Musts	32–44	1.89–2.15
Wines	n.d.	n.d.

## Data Availability

The data used to support the findings of this study can be made available by the corresponding author upon request.

## Supplementary Materials

The following supporting information can be downloaded at: https://www.mdpi.com/article/10.3390/foods12163091/s1, Table S1. List of the grape varieties used; Table S2. The assay codes and IDs, the DNA sequences surrounding the target polymorphisms, the Allele-Specific Primers (ASPs), and the Locus-Specific Primers (LSPs) are reported.
