# Supplementary material for: A Molecular Toolbox to Identify and Quantify Grape Varieties: On the Trace of “Glera”
Source: Foods. 2023 Aug 17;12(16):3091. doi: 10.3390/foods12163091 (PMC10453920; doi:10.3390/foods12163091)
Supplement: Supplementary file 1 [file foods-12-03091-s001.zip › Table S2.pdf]

Table S2

ASSAY CODE	ASSAY_ID	SNP_NAME	ALLELE	ASP1_NAME	ASP2_NAME	SNP_SEQ	ASP1_SEQ	ASP2_SEQ	LSP_SEQ
1	GTA0335201	VIT_1_10587347	CA	C	A	AAGGAGAGCATTTGGTTCTCTTCC[C/A]ATTATGGATTTGATGGAGCTTTCATTGGG	AAGGAGAGCATTTGGTTCTCTTCC	GAAGGAGAGCATTTGGTTCTCTTCA	CCCAATGAAAGCTCCATCAAATCCATAAT
2	GTA0335202	VIT_1_22859684	TG	T	G	ATCTTCTACTCATCGTTTTCCTTCCAA[T/G]TTCCTTCCCTCATTTTTCTTACTATTATTTATATA	ATCTTCTACTCATCGTTTTCCTTCCAA	CTTCTACTCATCGTTTTCCTTCCAC	TATATAAATAATAGTAAGAAAAATGAGGGAAGGAA
3	GTA0335178	VIT_1_729514	AG	A	G	GCTGAAGGTTTCTTGAAAAAGTACTGAT[A/G]AATTCCCTTTGCTTGTCCAACCTGTG	GCTGAAGGTTTCTTGAAAAAGTACTGAT	CTGAAGGTTTCTTGAAAAAGTACTGAC	CACAGGTTGGACAAGCAAAGGGAATT
4	GTA0335188	VIT_10_11783207	AT	A	T	TTCTGGCCTTTGAACCGCCCTA[A/T]TTGACAATTGAGGCTTTTCTCTCCTACT	TTCTGGCCTTTGAACCGCCCTA	TTCTGGCCTTTGAACCGCCCTT	AGTAGGAGAGAAAAGCCTCAATTGTCAA
5	GTA0335215	VIT_11_16594893	CT	C	T	GATAAGTCCAGGATCCAGAGCC[C/T]TAGGAGTAACATGGCCACTCCCATA	GATAAGTCCAGGATCCAGAGCC	AAAGATAAGTCCAGGATCCAGAGCT	TATGGGAGTGGCCATGTTACTCCTA
6	GTA0335216	VIT_11_19390306	GA	G	A	GTTCAAGCTGGGGGAGAATATATAC[G/A]TAACGGCGATACATGGACTCCGAC	GTTCAAGCTGGGGGAGAATATATAC	GGTTCAAGCTGGGGGAGAATATATAT	GTCGGAGTCCATGTATCGCCGTTA
7	GTA0335218	VIT_12_19886382	TC	T	C	AAAATCTCCAGGGCCTTTCATCACA[T/C]TAGTAAGTGCAGCCTGCCAAATCAG	AAAATCTCCAGGGCCTTTCATCACA	ATCTCCAGGGCCTTTCATCACG	CTGATTTGGCAGGCTGCACTTACTA
8	GTA0335189	VIT_12_22228357	AC	A	C	CAACTTCACGGTGACCGAATTCATA[A/C]AAACCTGTGGCTGCCGTCGACC	CAACTTCACGGTGACCGAATTCATA	AACTTCACGGTGACCGAATTCATC	GGTCGACGGCAGCCACAGGTTT
9	GTA0335217	VIT_12_739916	CT	C	T	GCTGAACACACTTTTTCCAAGTTCG[C/T]AATTAGATCTTTGTCCCCTCCCAAACC	GCTGAACACACTTTTTCCAAGTTCG	AAGCTGAACACACTTTTTCCAAGTTCA	GGTTTGGGAGGGGACAAAGATCTAATT
10	GTA0335190	VIT_13_15071171	AT	A	T	AGGTGGTTGTGCTGAGATGGCA[A/T]ATAGACCATCTGCCTTGTATTTCTCACT	AGGTGGTTGTGCTGAGATGGCA	AGGTGGTTGTGCTGAGATGGCT	AGTGAGAAATACAAGGCAGATGGTCTAT
11	GTA0335219	VIT_13_16003753	AG	A	G	ACTAAAATCTCCCACAGCTTCCTCA[A/G]TAGTAATTTATGTGGCTCGTAATCATGACC	ACTAAAATCTCCCACAGCTTCCTCA	TAAAATCTCCCACAGCTTCCTCG	GGTCATGATTACGAGCCACATAAATTACTA
12	GTA0335192	VIT_14_26212859	GA	G	A	GACTGAATTAGGGGGGTTTGGG[G/A]TAGCTTCCAATAACATGGATGACCACG	GACTGAATTAGGGGGGTTTGGG	CGACTGAATTAGGGGGGTTTGGA	CGTGGTCATCCATGTTATTGGAAGCTA
13	GTA0335193	VIT_14_27971842	AG	A	G	TGTTATTGAACAATCAGTGAGTTGGGT[A/G]AAGCATAAAAAAATGGTACTTGTTGCACCAT	TGTTATTGAACAATCAGTGAGTTGGGT	GTTATTGAACAATCAGTGAGTTGGGC	ATGGTGCAACAAGTACCATTTTTTTATGCTT
14	GTA0335191	VIT_14_4947068	TG	T	G	TATTCCTACCTGCGTTTCGTCGA[T/G]AAGCCCCACCGGGCCACAG	TATTCCTACCTGCGTTTCGTCGA	ATTCCTACCTGCGTTTCGTCGC	CTGTGGCCCGGTGGGGCTT
15	GTA0335194	VIT_15_18127737	TG	T	G	GGATGTGATGTAGGGGTCCTTGT[T/G]TTGGGTTTTAGTCCTAGAAAAGGTGATATG	GGATGTGATGTAGGGGTCCTTGT	GATGTGATGTAGGGGTCCTTGG	CATATCACCTTTTCTAGGACTAAAACCCAA
16	GTA0335195	VIT_15_18567587	TG	T	G	CTGTATTAAAGCTCTTGGGTGAAAAACT[T/G]AATCTTGAGCGTATTTGTGCCCAACAAT	CTGTATTAAAGCTCTTGGGTGAAAAACT	TGTATTAAAGCTCTTGGGTGAAAAACG	ATTGTTGGGCACAAATACGCTCAAGATT
17	GTA0335196	VIT_16_11542284	AG	A	G	GTTCAACCAAAGCCTAACAATAGATCAT[A/G]AAAAAAGAGTTACAATACCTACCTCAACCC	GTTCAACCAAAGCCTAACAATAGATCAT	TTCAACCAAAGCCTAACAATAGATCAC	GGGTTGAGGTAGGTATTGTAACTCTTTTTT
18	GTA0335197	VIT_16_16198599	AG	A	G	GCACAACAATTTCTCCAGCTTCGTT[A/G]AACTTAACACCTACGTGAGTCCATTTCT	GCACAACAATTTCTCCAGCTTCGTT	CACAACAATTTCTCCAGCTTCGTC	AGAAATGGACTCACGTAGGTGTTAAGTT
19	GTA0335198	VIT_16_17950801	AG	A	G	TCTGCTTGAGGGGGAGTGACAA[A/G]TATGAATCAAGTGGGAGATGTTTCCACTA	TCTGCTTGAGGGGGAGTGACAA	CTGCTTGAGGGGGAGTGACAG	TAGTGGAAACATCTCCCACTTGATTCATA
20	GTA0335220	VIT_16_21202286	AG	A	G	ACCCAATGGAATCGATCCAATGTCA[A/G]TATACTTGGCTTCGTCTGAAATTAACCAG	ACCCAATGGAATCGATCCAATGTCA	CCAATGGAATCGATCCAATGTCG	CTGGTTAATTTCAGACGAAGCCAAGTATA
21	GTA0335199	VIT_17_8729316	TA	T	A	CCATTTTTTGACTTTTGGGTTTACTTTGTT[T/A]TTTTATTGATGCTGTCCTCTTTATCATCCTA	CCATTTTTTGACTTTTGGGTTTACTTTGTT	CCATTTTTTGACTTTTGGGTTTACTTTGTA	TAGGATGATAAAGAGGACAGCATCAATAAAA
22	GTA0335200	VIT_18_11138668	AG	A	G	GACATGTTTCCATACTGATCCTCCTA[A/G]ATCATCATCATCATGGTTGAAAATGTCCAG	GACATGTTTCCATACTGATCCTCCTA	ACATGTTTCCATACTGATCCTCCTG	CTGGACATTTTCAACCATGATGATGATGAT
23	GTA0335221	VIT_18_1143527	AT	A	T	ACCATTCGTCCAAATGCCAAAAAACTA[A/T]TTGTTCCTGAATAAAGGTAACACGAGTTG	ACCATTCGTCCAAATGCCAAAAAACTA	ACCATTCGTCCAAATGCCAAAAAACTT	CAACTCGTGTTACCTTTATTCAGGAACAA
24	GTA0335222	VIT_19_4551426	AG	A	G	CAATGTGTATTACTTCAGTTTTGAACTCAA[A/G]TAAGTCCTTGTCTGGAATCTTTTGACAG	CAATGTGTATTACTTCAGTTTTGAACTCAA	AATGTGTATTACTTCAGTTTTGAACTCAG	CTGTCAAAAGATTCCAGACAAGGACTTA
25	GTA0335223	VIT_19_7217380	AT	A	T	GGTCTTCATCCAAGATATGTATCTCATT[A/T]AACTGCCCGCATCCATTTAATCGTATG	GGTCTTCATCCAAGATATGTATCTCATT	GGTCTTCATCCAAGATATGTATCTCATA	CATACGATTAAATGGATGCGGGCAGTT
26	GTA0335206	VIT_2_17198115	GC	G	C	TCCTCCGCTTCTCCGCCC[G/C]ATCTCGACTACGCCCGCAAGATTTT	TCCTCCGCTTCTCCGCCC	TCCTCCGCTTCTCCGCCG	AAAATCTTGCGGGCGTAGTCGAGAT
27	GTA0335203	VIT_2_2329515	AG	A	G	GTCCAGTTGTGGATTTCTCAGCAA[A/G]AACAAGAGAGGGAAGAAGAGCCCTT	GTCCAGTTGTGGATTTCTCAGCAA	TCCAGTTGTGGATTTCTCAGCAG	AAGGGCTCTTCTTCCCTCTCTTGTT
28	GTA0335204	VIT_2_4121375	GC	G	C	TGGATGCACGGATCATTGCTCC[G/C]ATTCTCATAATCATAGACCGTAAGAAGATTTTG	TGGATGCACGGATCATTGCTCC	TGGATGCACGGATCATTGCTCG	CAAAATCTTCTTACGGTCTATGATTATGAGAAT
29	GTA0335205	VIT_2_6863000	GA	G	A	AAAAAGAAAAGGGAAAACAAAAAACTACTCC[G/A]TTCAAACCAAACCCAACTAATCAATGAAATC	AAAAAGAAAAGGGAAAACAAAAAACTACTCC	AAAAAAAGAAAAGGGAAAACAAAAAACTACTCT	GATTTCATTGATTAGTTGGGTTTGGTTTGAA
30	GTA0335207	VIT_3_10039665	CA	C	A	GCGTGCCGGTTGAAATTGAATTTATG[C/A]AAATTTGTCTTTTGAGAGTTAAAACATGGAAACC	GCGTGCCGGTTGAAATTGAATTTATG	GCGTGCCGGTTGAAATTGAATTTATT	GGTTTCCATGTTTTAACTCTCAAAAGACAAATTT
31	GTA0335179	VIT_3_19173971	TA	T	A	CAAGTGACCAATCATGGAGTTCCAT[T/A]TTGCTACACCGCATGAGGAGCG	CAAGTGACCAATCATGGAGTTCCAT	CAAGTGACCAATCATGGAGTTCCAA	CGCTCCTCATGCGGTGTAGCAA
32	GTA0335181	VIT_5_11514532	CT	C	T	AATAAGGCAATTAACTTGACCAAGACG[C/T]TAACTGCTAAATTCCACAGACAAATGTAAAG	AATAAGGCAATTAACTTGACCAAGACG	GAATAAGGCAATTAACTTGACCAAGACA	CTTTACATTTGTCTGTGGAATTTAGCAGTTA
33	GTA0335208	VIT_5_1785979	CT	C	T	GGTCCCTCACCTATTACTCCAG[C/T]AAGAAACTCAAGCTTTCCATGTCCGAAA	GGTCCCTCACCTATTACTCCAG	CGGTCCCTCACCTATTACTCCAA	TTTCGGACATGGAAAGCTTGAGTTTCTT
34	GTA0335180	VIT_5_5773320	CT	C	T	GGCTGTATCCTGTCTTGAAGCTC[C/T]ATCATAAACAGCTCGTGACCTTGGATC	GGCTGTATCCTGTCTTGAAGCTC	TGGCTGTATCCTGTCTTGAAGCTT	GATCCAAGGTCACGAGCTGTTTATGAT
35	GTA0335209	VIT_5_6744629	AG	A	G	TGAGTCAATCTCGCCGAATATGAGT[A/G]AATATGGATATTGCTGTCCAATCTTGAGTTT	TGAGTCAATCTCGCCGAATATGAGT	AGTCAATCTCGCCGAATATGAGC	AAACTCAAGATTGGACAGCAATATCCATATT
36	GTA0335210	VIT_6_1032391	GA	G	A	TTACAACAGACGCTTTCTCCATGG[G/A]TTTCCCTCCTAATCTGACAACCCTAC	TTACAACAGACGCTTTCTCCATGG	GTTACAACAGACGCTTTCTCCATGA	GTAGGGTTGTCAGATTAGGAGGGAAA
37	GTA0335183	VIT_6_17593092	AT	A	T	CTGCATTCGTTCACCTGTCAACTTA[A/T]AATTAAGGACAAGCTCGAAGATGCAGAT	CTGCATTCGTTCACCTGTCAACTTA	CTGCATTCGTTCACCTGTCAACTTT	ATCTGCATCTTCGAGCTTGTCCTTAATT
38	GTA0335182	VIT_6_4258638	CT	C	T	TGTCCAGAGATCCTGTTTTCTCG[C/T]AACTTTCCATTGCTGCCTGCTGAAAG	TGTCCAGAGATCCTGTTTTCTCG	ACTGTCCAGAGATCCTGTTTTCTCA	CTTTCAGCAGGCAGCAATGGAAAGTT
39	GTA0335211	VIT_7_1388822	AG	A	G	CGTCCGGATGCATTGCGCCT[A/G]TACTCCAGCCAATCAGCGTTTGCC	CGTCCGGATGCATTGCGCCT	GTCCGGATGCATTGCGCCC	GGCAAACGCTGATTGGCTGGAGTA
40	GTA0335212	VIT_7_17760363	CT	C	T	GGATTTATGGAGGAAACAAAGAAAACTG[C/T]ATCAATGACCGGCAGTTCCAGCAG	GGATTTATGGAGGAAACAAAGAAAACTG	GGATTTATGGAGGAAACAAAGAAAACTA	CTGCTGGAACTGCCGGTCATTGAT
41	GTA0335185	VIT_8_12622984	TG	T	G	GATGAATGATTGCTAATTATATTTTAGCATCTA[T/G]TTAACGTCCACAGATAAATCACAATCAGAC	GATGAATGATTGCTAATTATATTTTAGCATCTA	ATGAATGATTGCTAATTATATTTTAGCATCTC	GTCTGATTGTGATTTATCTGTGGACGTTAA
42	GTA0335213	VIT_8_3320936	CT	C	T	TGGAGGGTAAAAATGAACTCAATTTGAC[C/T]TTCCCACTGCTTCTATCATCAGAAAGG	TGGAGGGTAAAAATGAACTCAATTTGAC	ATTGGAGGGTAAAAATGAACTCAATTTGAT	CCTTTCTGATGATAGAAGCAGTGGGAA
43	GTA0335184	VIT_8_7904164	CG	C	G	TCCAATTTTCAATTCATTTAAGAGCATGAC[C/G]ATCAAAGTTTCAATTCAGATCCGAGGTAG	TCCAATTTTCAATTCATTTAAGAGCATGAC	TCCAATTTTCAATTCATTTAAGAGCATGAG	CTACCTCGGATCTGAATTGAAACTTTGAT
44	GTA0335187	VIT_9_16237722	GA	G	A	GCAAGTCGAGCTCTCTGTCCG[G/A]AAGTTGACTCCTTTATCTCCAATATAAGCC	GCAAGTCGAGCTCTCTGTCCG	AGCAAGTCGAGCTCTCTGTCCA	GGCTTATATTGGAGATAAAGGAGTCAACTT
45	GTA0335186	VIT_9_3123999	AT	A	T	TGTCTTGACTCATCATATTTGACAGCA[A/T]ATTGGACCCATTATCGTCTTCACACC	TGTCTTGACTCATCATATTTGACAGCA	TGTCTTGACTCATCATATTTGACAGCT	GGTGTGAAGACGATAATGGGTCCAAT
46	GTA0335214	VIT_9_6730580	GT	G	T	TGTTGCAGCAGATAATGCATACGAC[G/T]AATAAAGGTGCTGATGATAAAATAGAAGTGTG	TGTTGCAGCAGATAATGCATACGAC	ATGTTGCAGCAGATAATGCATACGAA	CACACTTCTATTTTATCATCAGCACCTTTATT
